# Supplementary material for: Endometrial cells with high ALDH activity contribute to uterine development and regeneration
Source: eLife. 2026 Aug 6;15:RP110975. doi: 10.7554/eLife.110975 (PMC13446906; doi:10.7554/eLife.110975)
Supplement: Supplementary file 4 [file elife-110975-supp4.docx]

**Supplementary File 4. List of primers and antibodies**

| **Genotyping Primers** | **Forward Primer (5’-3’)** | **Reverse Primer (5’-3’)** |
| --- | --- | --- |
| *Aldh1a1^creERT2^* (promoter) | AGTCTGCCCATCCAATCATATC |  |
| *Aldh1a1^creERT2^* (*Aldh1a1*) | TCTATGGTTCACTGCACTCTGG |  |
| *Aldh1a1^creERT2^* (Cre) | GTTCTTGCGAACCTCATCACTC |  |
| Ai9 LSL-tdTomato (oIMR9020) | AAGGGAGCTGCAGTGGAGTA |  |
| Ai9 LSL-tdTomato (oIMR9021) | CCGAAAATCTGTGGGAAGTC |  |
| Ai9 LSL-tdTomato (oIMR9103) | GGCATTAAAGCAGCGTATCC |  |
| Ai9 LSL-tdTomato (oIMR9105) | CTGTTCCTGTACGGCATG G |  |
| DTR (mutant) |  | GCGAAGAGTTTGTCCTCAACC |
| DTR (common) | AAAGTCGCTCTGAGTTGTTAT |  |
| DTR (WT) |  | GGAGCGGGAGAAATGGATATG |
| **PCR Primers** | **Forward Primer (5’-3’)** | **Reverse Primer (5’-3’)** |
| *Aldh1a1* | ATACTTGTCGGATTTAGGAGGCT | GGGCCTATCTTCCAAATGAACA |
| *Gapdh* | CAATGTGTCCGTCGTGGATCT | GCCTGCTTCACCACCTTCTT |
| *Rpl17* | ACCGCACTGAGATTCGGATG | GAACCTTACGAACCATTTGGGC |
| *Hprt* | GTGATTAGCGATGATGAACCA | GCAAGTCTTTCAGTCCTGTC |
| **Antibody Name** | **Vendor, Catalog#, RRID** | **Dilution used** |
| ALDH1A1 | Abcam, ab52492, AB_867566 | 1:50 (IHC) |
| Ki-67 | BD Pharmingen, 550609, AB_393778 | 1:500 (IHC) |
| cleaved caspase-3 | Cell Signaling, 9661, AB_2341188 | 1:400 (IHC) |
| RFP | Rockland, 600-401-379, AB_2209751 | 1:500 (IHC) |
| RFP | Abcam, ab125244, AB_10973556 | 1:200 (IF) |
| FOXA2 | Abcam, ab108422, AB_11157157 | 1:200 (IHC), 1:50 (IF) |
| Cytokeratin 8 | DSHB, TROMA-1, AB_531826 | 1:50 (IF) |
| Vimentin | Cell Signaling, 5741, AB_10695459 | 1:200 (IF) |
| Phospho Histone H3 | Abcam, ab5176, AB_304763 | 1:200 (IF) |
| Acetyl-α-Tubulin | Cell Signaling, 5335, AB_10544694 | 1:200 (IF) |
| Anti-Rabbit IgG Antibody | Vector Laboratories, BA-1000, AB_2313606 | 1:200 (IHC) |
| Anti-Mouse IgG Antibody | Vector Laboratories, BA-9200, AB_2336171 | 1:200 (IHC) |
| Anti-Rat IgG (H+L), AF488 | Invitrogen, A-21208 | 1:250 (IF) |
| Anti-Rabbit IgG (H+L), AF594 | Invitrogen, A-21207 | 1:250 (IF) |
| Anti-Mouse IgG (H+L), AF488 | Invitrogen, A-21202 | 1:250 (IF) |
| Anti-Rabbit IgG (H+L), AF568 | Invitrogen, A-10042 | 1:250 (IF) |
| Anti-Rat IgG (H+L), AF647 | Invitrogen, A78947 | 1:250 (IF) |
