# Supplementary material for: Endometrial cells with high ALDH activity contribute to uterine development and regeneration
Source: eLife. 2026 Aug 6;15:RP110975. doi: 10.7554/eLife.110975 (PMC13446906; doi:10.7554/eLife.110975)
Supplement: Peer review history

# Editor report: eLife Assessment

**DOI:** 10.7554/eLife.110975.3.sa0

**Authors:** Wei Yan

**Affiliations:** Washington State University United States

**Keywords:** Convincing, Valuable

This **valuable** study reports that ALDH-abundant cells exhibit stem cell properties and may play a key role in endometrial epithelial development in mice. The data support the main conclusion and are **convincing**. This work will be of great interest to reproductive biologists and biomedical researchers working on women's reproductive health.

# Referee report: Reviewer #1 (Public review)

**DOI:** 10.7554/eLife.110975.3.sa1

The manuscript by Tang et al. characterizes the expression dynamics and functional roles of aldehyde dehydrogenase 1 activity in uterine physiology. Using a combination of in vivo lineage tracing and cell ablation coupled with organoid culture, the authors propose that Aldh1a1 lineage-marked cells contribute to uterine gland development and epithelial regeneration. The descriptive data will be of interest to reproductive biologists and clinicians and builds on established hypotheses in the field. The manuscript is well written and scientifically sound and the important experimental limitations and interpretation caveats are presented throughout.

The majority of my initial comments have been adequately addressed within the text.

Remaining limitations include:

(1) The impact of tamoxifen injection directly on Aldh1a1 expression in the developing uterus.

(2) It would be beneficial to demonstrate the degree of cell death following diphtheria toxin treatment 24-48 hours after injection in Tam-treated mice at PND 10. It is not clear as to why the 4-day timepoint was selected. Cells expressing the DTR should begin undergoing apoptosis within several hours after treatment.

# Referee report: Reviewer #2 (Public review)

**DOI:** 10.7554/eLife.110975.3.sa2

Tang et al. investigated the contribution of Aldh1a1+ cells, as putative stem/progenitor cells, to endometrial development, maintenance during the estrous cycle, and postpartum repair in mouse models. They employed in vitro organoid formation and in vivo lineage tracing models coupled with RNA-seq to test the stem-ness of Aldh1a1+ cells. They found that mouse endometrial cells with high ALDH activity (using the ALDEFLUOR assay) formed more and larger organoids and were enriched for stem/progenitor cell gene signatures. Similar results were shown using endometrial cells from a human patient sample. Epithelial ALDH1A1 expression was shown to be hormonally regulated, becoming more restricted to the glands, a putative epithelial stem cell niche, under estrogen stimulation. Using lineage-tracing initiated postnatally/prepubertally, Aldh1a1+ epithelial cells were shown to expand, contributing to both the luminal and glandular epithelium into adulthood, whereas adult initiation of labeling showed expansion of stromal Aldh1a1+ cells but not epithelial. Postnatal ablation of single-labeled Aldh1a1+ epithelial cells resulted in impaired gland development. Lastly, Aldh1a1-lineage traced cells (adult labeled) were present during postpartum endometrial repair as were epithelial/mesenchymal transitional cells.

Comments on revised version.

In the revised manuscript, comments have largely been addressed and the manuscript is improved. The authors have tempered their inference of the contribution of ALDH1A1+ cells to endometrial regeneration, but the conclusions are still somewhat overstated. However, the overall work provides valuable insight and strengthens the growing body of literature characterizing endometrial stem/progenitor cells and their function.

# Referee report: Reviewer #3 (Public review)

**DOI:** 10.7554/eLife.110975.3.sa3

Summary:

Tan et al demonstrated the importance of ALDH-high cells in the epithelial development in the mouse endometrium, and these cells displayed properties of stem cells.

Strengths:

The findings are solid, supported and validated through a combination of technical methods. I appreciated this combined use of mouse and human endometrial cells to strengthen the findings. Genomic results from a single-cell sequencing dataset were informative as they depicted the different stages of the estrus cycle during the regeneration process. Verification with immunostainings with various markers made it convincing for readers to visualize the cell's location, progression, and status at different timepoints. Utilizing human endometrial cells further demonstrated that the phenomenon observed in mice can be translated to humans.

This work will greatly advance the understanding of endometrial regeneration for reproductive biologists.

Comments on revised version.

The authors have answered the questions in the revised manuscript, no further comments.

# Author comment: Author response

**DOI:** 10.7554/eLife.110975.3.sa4

**Authors:** Suni Tang, Anna Catherine Unser, Peixin Jiang, Sydney E Parks, Genesis J Herrera, Ting Geng, Linda Alpuing Radilla, Brooke A Thigpen, Xiaoming Guan, Diana Monsivais

**Affiliations:** Baylor College of Medicine Houston United States; Baylor College of Medicine Houston United States; Baylor College of Medicine Houston United States; Baylor College of Medicine Houston United States; Baylor College of Medicine Houston United States; Baylor College of Medicine Houston United States; Baylor College of Medicine Houston United States; Baylor College of Medicine Houston United States; Baylor College of Medicine Houston United States; Baylor College of Medicine Houston United States

The following is the authors’ response to the original reviews

**eLife Assessment**

This valuable study reports that the ALDH-abundant cells display stem cell properties and may play a key role in the endometrial epithelial development in the mouse. The data supporting the main conclusion are solid, although further improvements are needed to strengthen the conclusions. This work will be of great interest to reproductive biologists and biomedical researchers working on women's reproductive health.

We thank the reviewers and editor for their critical reading and assessment of our manuscript. We carefully considered each of the points raised by the reviewers. In this document and in the edited manuscript and figures, we have carefully addressed each of the comments and requested modifications. In light of these changes, we expect that you will find that the manuscript has improved.

We indicate our responses to the reviewers below in blue font and highlight the changes in the manuscript using the line numbers corresponding to the tracked version of the revised document.

**Public Reviews:**

**Reviewer #1 (Public review):**

The manuscript by Tang et al. characterizes the expression dynamics and functional roles of aldehyde dehydrogenase 1 activity in uterine physiology. Using a combination of in vivo lineage tracing and cell ablation coupled with organoid culture, the authors propose that Aldh1a1 lineage-marked cells contribute to uterine gland development and epithelial regeneration. The descriptive data will be of interest to reproductive biologists and clinicians and will build on established hypotheses in the field. The manuscript is well written and scientifically sound; however, several experimental limitations and interpretation caveats should be addressed.

We thank the reviewer for their comments and expert assessment of our paper.

(1) The methods surrounding the passage number and duration of culture following sorting prior to transcriptomic profiling should be clarified in the figure legends. Related to this, the representative images in Figures 1D and 1E do not appear consistent with the quantification presented in Figures 1F-H and should be reconciled.

Thanks for this comment. We have now clarified this in the Figure 1 legend as follows,

LINES 1026-1029: “Organoid formation assay performed immediately after luminal epithelial cell isolation and by plating equal numbers of viable ALDH^LO^ (D) and ALDH^HI^ (E) epithelial cells. ALDH^LO^ and ALDH^HI^ organoids were cultured for two weeks and passaged once prior to the organoid formation assays and transcriptomic analyses.”

Regarding the second comment, we recognize that the images we showed may not have been the most representative of our quantification. As such, we replaced them with the organoid images so that they better reflect the quantification outlined in Figure 1F-H.

(2) The conclusion that ALDH1A1+ cells are enriched in populations with stem cell characteristics relies primarily on transcriptomic analysis. Protein-level co-localization should be performed to strengthen this claim.

We thank the reviewer for this comment. Unfortunately, the antibodies for many of these stem cell markers (such as LGR5, AXIN2, and SUSD2) are not well-suited for immunostaining. Others that have been proposed in human and are amenable to immunostaining are not suitable markers for mouse endometrial stem cells (such as CDH2). We hope that by showing that ALDH1A1 is expressed in patterns that are similar to the previously published stem cell markers LGR5 and AXIN2 (i.e., throughout the epithelium in the developing uterus and subsequently enriched in the tips of the endometrial glands of adult mice), along with transcriptomic studies, we can demonstrate its utility as a marker for mouse endometrial stem cells.

(3) The overlap of 19 genes between the data set here and AXIN2 HI data is presented as evidence of shared stemness identity, but no statistical assessment of this overlap is provided. A hypergeometric test should be performed to determine whether this overlap is greater than expected by chance.

Thank you for this suggestion. We have performed a hypergeometric test and determined that the reported shared genes between the two datasets are greater than is expected by chance. We have updated the results section to state the following:

Lines 137-140: "We determined that the overlap between ALDH^HI^ and Axin2^+^ stemness marker genes was significantly greater than expected by chance for both upregulated (21/346 genes, 1.81-fold enrichment, p = 0.0067) and downregulated (19/674 genes, 1.67-fold enrichment, p = 0.021) gene sets (hypergeometric test, universe = 23,182 genes)."

(4) The impact of tamoxifen injection on Aldh1a1 expression should be characterized in the neonatal uterus, as tamoxifen itself has known estrogenic activity that could confound interpretation of the lineage tracing results at early postnatal timepoints.

Although we took measures to control for this possibility by using multiple time-points and models to trace the impact of *Aldh1a1*^+^ cells in development and adulthood, we recognize the importance of this comment and acknowledge that this is a limitation in the design of our study. We have included the following text to the Discussion acknowledging this point:

Lines 433-441: “Given the well-documented impacts of tamoxifen for lineage tracing studies, it is imperative to use doses of tamoxifen that will minimize estrogenic impacts and result in off-target effects (Rios et al., 2016). This often requires administration at doses that will achieve maximal recombination of the desired gene, while ensuring that the potential deleterious impacts of tamoxifen are minimized (Chen et al., 2023; Pimeisl et al., 2013). The cre/ERT2 tamoxifen inducible model is widely used to study uterine biology where it serves as a useful tool to interrogate the spatiotemporal impact of key genes, either through inactivation or for lineage tracing. Despite its widely documented utility across many tissue types and developmental timepoints, the use of tamoxifen and its impacts on the endometrium remain a limitation of our study, which we tried to address by implementing multiple timepoints, doses, and orthogonal assays in our experimental design.”

(4b) Related to this, while low-dose tamoxifen is shown to label individual cells within 24 hours of injection, the translation dynamics of the label following Cre-mediated recombination can require up to 72 hours. The presence of only a few labeled clones at PND8 but multiple separate clones per cross-section at later timepoints warrants discussion and may reflect labeling kinetics rather than clonal expansion.

The reviewer raises an important point. We agree that the 72hr-translation kinetics of the cre-mediated recombination is a legitimate consideration for interpreting our data and we have added the text below to the Discussion section acknowledging this point.

We have addressed this by adding the following text to the discussion:

Lines 417-422: We hypothesized that the singly labeled cells observed from one day tracing experiments expanded in a clonal fashion during the various timepoints we measured. We note that the translation kinetics of the labeled cells following cre-mediated recombination may contribute to the limited labeling observed at PND8/PND15 and there is a potential for delayed labeling of cells between 24 and 72 hours of tamoxifen administration. However, the continuous increase in labeled cells at the subsequent timepoints favors our interpretation of clonal expansion as the primary explanation.

(5) It would strengthen the in vivo ablation data to validate the degree of cell death following diphtheria toxin treatment directly. It is possible that a general decrease in cell number rather than specific loss of a stem cell population is responsible for the observed reduction in gland number and FOXA2 expression (Tongtong et al 2017).

We agree that this is an important control to incorporate into our experimental design. To rule out this possibility, we performed immunohistochemistry of cleaved caspase 3 in the uterine tissues of DTR^flox/flox^ and *DTR^flox/flox^*;*Aldh1a1*^cre/ERT2^ mice 4 days after administration of diphtheria toxin. The results indicate similar levels of cleaved caspase 3 detection in both genotypes, suggesting that the decrease in FOXA2+ cells is not due to non-specific cell death, but rather the result of ALDH1A1^+^ cells. These data and the following text have been added to the manuscript:

Lines 320-324: “We determined that the decreased in FOXA2^+^ cells in the experimental mice was not the result of non-specific DT-mediated cell death, as similar levels of cleaved caspase 3-positive cells were detected in the DT-treated control *ROSA26*^DTR/DTR^ and *ROSA26*^DTR/DTR^;*Aldh1a1*^cre/ERT2/+^ mice 4 days post-diphtheria toxin administration (Figure S3G-H’).”

(6) The lineage tracing data in the postpartum endometrium demonstrate that Aldh1a1-marked cells are present during regeneration, but it remains unclear whether these cells are preferentially activated or expanded in response to tissue injury. Coupling these studies with diphtheria toxin-mediated ablation during active regeneration would more directly test the proposed regenerative role of this population.

This is a great point and one that we would be very interested in pursuing as follow-up studies in our future work. Regretfully, due to the long generation time and experimental procedures associated with these proposed studies, we are not able to include these experiments in the current manuscript. Thus, we have changed our wording and conclusions throughout the manuscript to be less definitive in terms of the role of Aldh1a1 in regeneration, since this will be the focus of future studies.

The contribution of stromal Aldh1a1 lineage-positive cells is underexplored in the discussion, given the lineage tracing data showing stromal labeling across multiple timepoints and its potential relevance to mesenchymal-to-epithelial transition.

Thank you for the suggestion. We have now expanded this section in the Discussion to include the following:

Lines 496-504: We also found ALDH1A1^+^ stromal cells were more prevalent when tracing began in adult mice. Other studies have shown that mesenchymal cells contribute to endometrial regeneration in the postpartum phase or after induced menses through a process of MET (Cousins et al., 2014; Kirkwood et al., 2022; Li et al., 2025). Similarly, lineage tracing studies have shown that MET is an active process and contributes to epithelial cell regeneration in the post-partum phase (Huang et al., 2012; Patterson et al., 2013). Although this is an area of active investigation in the field, with some contradicting reports, it is plausible to hypothesize that endometrial tissue has the capacity to undergo wound-healing and regeneration via several mechanisms (Ang et al., 2023; Ghosh et al., 2020). The process of MET in wound healing is widely documented in other organs, such as the kidney, liver and lung, where MET is associated with depletion of the resident epithelial cell pool (Bi et al., 2012; Niayesh-Mehr et al., 2024; Zeisberg et al., 2005).

Finally, the word 'control' may overstate the functional evidence presented. 'Contribute' may be more accurate given the partial and context-dependent nature of the phenotypes observed.

We agree with the reviewer’s point that control may overstate the evidence that we provide in the manuscript. To reflect this, we have edited the manuscript title and text to address this suggestion.

**Reviewer #2 (Public review):**

Tang et al. investigated the contribution of Aldh1a1+ cells, as putative stem/progenitor cells, to endometrial development, maintenance during the estrous cycle, and postpartum repair in mouse models. They employed in vitro organoid formation and in vivo lineage tracing models coupled with RNA-seq to test the stem-ness of Aldh1a1+ cells. They found that mouse endometrial cells with high ALDH activity (using the ALDEFLUOR assay) formed more and larger organoids and were enriched for stem/progenitor cell gene signatures. Similar results were shown using endometrial cells from a human patient sample. Epithelial ALDH1A1 expression was shown to be hormonally regulated, becoming more restricted to the glands, a putative epithelial stem cell niche, under estrogen stimulation. Using lineage-tracing initiated postnatally/prepubertally, Aldh1a1+ epithelial cells were shown to expand, contributing to both the luminal and glandular epithelium into adulthood, whereas adult initiation of labeling showed expansion of stromal Aldh1a1+ cells but not epithelial. Postnatal ablation of single-labeled Aldh1a1+ epithelial cells resulted in impaired gland development. Lastly, Aldh1a1-lineage traced cells (adult labeled) were present during postpartum endometrial repair as were epithelial/mesenchymal transitional cells.

This study addresses an important area of research in the field of endometrial stem/progenitor cell biology. The authors are commended for their use of multiple complementary methods, including lineage tracing, DTR-mediated cell ablation, organoid assays, and RNA-seq in mouse and human models to assess the stem-like nature of Aldh1a1+ cells. The data support the stem/progenitor phenotype of Aldh1a1+ epithelial cells during endometrial development; however, there are noted discrepancies between organoid formation assays and lineage tracing experiments regarding the stemness of Aldh1a1+ epithelial cells in adults. Specifically, organoids were generated from adult cells and demonstrated in vitro stem cell activity; however, in vivo lineage-tracing of adult cells either during the estrous cycle or postpartum repair does not show expansion of Aldh1a1+ cells, suggesting they do not have stem/progenitor activity. Additionally, the stem-ness of epithelial vs stromal Aldh1a1+ cells is confounded in the study because epithelial cells were not purified for organoid experiments, epithelial cells were not exclusively lineage-traced as stromal cells were also labeled, and mesenchymal-epithelial transition was suggested to occur during postpartum repair. The following specific comments are presented to detail these concerns:

We thank the reviewer for their critical reading of our manuscript and constructive comments.

(1) The statement in the brief summary, "...critical for lifelong endometrial regeneration," is not supported by the data provided.

We have edited the brief summary to exclude this statement, it now reads as follows:

Lines 4-5: “We uncover ALDH1A1^+^ cells as a group of hormone sensitive stem cells contributing to endometrial development and regeneration.”

(2) AlDH1A1 is not restricted to the endometrial epithelium, and epithelial cells were not purified by flow cytometry for experiments in Figure 1. Figure 2 clearly shows the presence of mesenchymal cells, even using the described method for enriching for epithelial cells. Therefore, contaminating mesenchymal cells with high ALDH activity may confound the experimental results in Figure 1, either through promoting epithelial cell growth or through MET. The authors should provide clear evidence of epithelial purity in organoid experiments or that mesenchymal cells are not contained in the ALDHhi population. These comments also apply to the human organoid experiments in Figure 7.

We thank the reviewer for raising this important point. Our group has been using the enzymatic method to routinely separate epithelial from stromal cell populations from the mouse uterus (see references dating back to 2015, PMID 26721398, 28324064, 34099644). In these experiments we typically obtain >98% purity in the epithelial and stromal cell compartments, respectively. We can directly observe this purity in the immunofluorescence images shown I Author response image 1 and Author response image 2, where mouse endometrial epithelial cells and stromal cells were enzymatically separated and immunostained with E-cadherin and vimentin antibodies to detect epithelial and mesenchymal cells in both cell preparations. The images show very few contaminating epithelial and stromal cells in either cell preparation. We have observed similar results when preparing epithelial and stromal cell preparation from the human endometrium, where the epithelial cell organoids display high purity with ~100% epithelial cell expression when we perform immunostaining.

**Author response image 1.** Purity of mouse endometrial epithelial cells obtained via enzymatic and mechanical dissociation. (A-B) Shows the epithelial (A) and stromal (B) cells plated on glass coverslips and immunostained with an epithelial cell marker (cytokeratin 8, red), a stromal cell marker (vimentin, green), and DAPI.

**Author response image 2.** Human endometrial epithelial organoids were fixed and immunostained with cytokeratin 8 (green) and DAPI. The images are typical for our epithelial cell cultures and demonstrate that all epithelial cells are CK8-positive.

(3) Lines 186-187: Susd2 was increased in EpSC clusters, yet this is a mesenchymal stem/progenitor marker in humans. The authors should discuss the implications of this.

We thank the reviewer for highlighting this. We have now included the following in our Discussion to address this point:

Lines 527-532: Clustering with this population of EpSCs were *Susd2*^+^ cells, which are well-characterized mesenchymal progenitors that are enriched in the perivascular regions of the human endometrium (Darzi et al., 2016; Khanmohammadi et al., 2021). The presence of *Susd2*^+^ cells, while unexpected in an epithelial stem cell niche, could indicate the presence of a transitional mesenchymal or perivascular cell that is differentiating into epithelium. Evidence for both mesenchymal and *Nestin2*^+^ pericytes have been recently described in the mouse endometrial epithelium (Kirkwood et al., 2022; Li et al., 2025).

(4) In Figure 5, RFP+ epithelial cells should be quantified as in previous figures to substantiate the statement in lines 279-280, "At PPD5, the proportion of RFP+ epithelial cells had expanded relative to PPD1 and PPD3 (Figure 5E-E')." Especially because in the low mag images (C-E), RFP+ epithelial cells appear to be most abundant at PPD1 and decrease at PPD3 and PPD5, suggesting that they may not be involved in endometrial regeneration/repair (contradicting the interpretation in line 285). Further, if there is in fact a decrease over postpartum repair, then regeneration should be removed from the title of the manuscript. RFP+ stromal cells should also be quantified.

We appreciate this reviewer’s comment and agree that as stated, the conclusion is not fully supported by the data. To address this comment, we have edited the results so that they clearly indicate the results and remove any ambiguity:

As requested, we quantified the number of RFP+ stromal and epithelial cells during the postpartum phase and noted that RFP+ cells were prominent in the stromal compartment of the endometrium. While RFP+ epithelial were also observed during these timepoints, they were less abundant than RFP+ stromal cells. Because the number of RFP+ cells did not significantly change over the postpartum phases in neither the stromal nor epithelial compartment, we have modified our conclusion to state that ALDH1A1+ cells are transiently detected in the regenerating endometrium.

Results:

Lines 287-294: “By analyzing the uterine tissues near the placental detachment site, we observed that RFP positive cells were prominent in the endometrial stromal cells that were adjacent to the luminal epithelium (Figure 5C-C’, green arrows). RFP^+^ cells were also observed in the stromal cells near the placental detachment sites at PPD1 and PPD3 (Figure 5D’-E’, red & blue arrows) and in limited luminal epithelial cells (Figure 5D”,E”). Quantification of RFP+ cells throughout these postpartum phases indicated that stromal cells had more frequent ALDH1A1^+^ stromal cells (360 ± 103, PPD1, n=3; 217 ± 107, PPD3, n=3; 254 ± 32, PPD5, n=4) than ALDH1A1^+^ epithelial cells in the regenerating endometrium (65 ± 65, PPD1, n=3; 20 ± 10, PPD3, n=3; 114.25 ± 39, PPD5, n=4) (Figure S4).”

Discussion:

Lines 512-520: “We also noted that a majority of ALDH1A1^+^ cells were localized to the active areas of endometrial regeneration near the placental detachment sites at PPD1 with a pronounced expression in the sub-epithelial stromal cells. As regeneration progressed, we continued to observe ALDH1A1^+^ cells in the stromal compartment within the placental detachment sites at PPD3 and PPD5, with a progressive, but not statistically significant, increase in ALDH1A1^+^ epithelial cells. Collectively, our data demonstrate that ALDH1A1^+^ lineage cells participate in the restoration of endometrial architecture and functional compartments in the postpartum phase, even if their direct contribution is transient. Future detailed and mechanistic studies will be necessary to fully characterize their role in this process and their long-term consequence in postpartum regeneration.”

(5) For Figure 7F, it should be clearly stated in the main text that the results are from one patient sample and the data presented are experimental replicates, so as not to be confused with biological replicates (the same for Supplementary Figure S4). Were B and G in Figure 7 also from one patient?

Thanks for pointing this out. We have edited the figure legends in the main text and supplemental figures to indicate this.

Lines 336-337: “…main figures show representative results from one patient sample performed in technical replicates, with additional patient samples included in the supplement…”

(6) Lines 425-427: "Ovariectomized mice treated with 90-day E2 pellets, on the other hand, showed a complete restriction of ALDH1A1 to the glandular crypts." In Figure 2 S' ALDH1A1+ cells are visible in the LE (the staining is lighter than in the GE but looks real), contradicting this statement.

This is an important distinction. We have now edited this part of the manuscript to state:

Lines 458-461: “Ovariectomized mice treated with 90-day E2 pellets, on the other hand, showed enriched ALDH1A1 in the glandular crypts with weak luminal epithelial staining, while the ovariectomized controls had strong ALDH1A1 expression throughout the luminal and glandular epithelium.”

(7) Lines 466-467: "In cycling mice, we found sporadic cells that expressed both stromal and epithelial markers in the ALDHA1+ cells." These data are not presented.

We apologize for the confusion, this sentence has been removed from the discussion.

(8) These data support the role of Aldh1a1+ cells in endometrial epithelial development, but conclusions about their role in repair/regeneration should be tempered as the data are much weaker here.

We thank the reviewer for their overall assessment. To address this point, we have thoroughly edited the appropriate areas to temper the conclusions and ensure that they are strongly supported by our data. We have also edited the manuscript’s title to reflect this.

**Reviewer #3 (Public review):**

Summary:

Tan et al demonstrated the importance of ALDH-high cells in the epithelial development in the mouse endometrium, and these cells displayed properties of stem cells.

We thank the reviewer for their assessment of our manuscript.

Strengths:

The findings are solid, supported and validated through a combination of technical methods. I appreciated this combined use of mouse and human endometrial cells to strengthen the findings. Genomic results from a single-cell sequencing dataset were informative as they depicted the different stages of the estrus cycle during the regeneration process. Verification with immunostainings with various markers made it convincing for readers to visualize the cell's location, progression, and status at different timepoints. Utilizing human endometrial cells further demonstrated that the phenomenon observed in mice can be translated to humans.

This work will greatly advance the understanding of endometrial regeneration for reproductive biologists.

We thank the reviewer for their expert assessment and positive comments regarding our manuscript.

Weaknesses:

No major weaknesses were identified by this reviewer.

**Recommendations for the authors:**

**Reviewer #2 (Recommendations for the authors):**

(1) As this study evaluated Aldh1a1+ cells in both the epithelium and stroma, it is recommended that the title and abstract be revised to reflect this.

Thank you. Both the title and abstract title have been updated to reflect this comment.

(2) Lines 46-47 in the abstract: "Aldh1a1+ cells expanded during postnatal development, estrus cycling, and following post-partum repair." It is recommended to clarify stromal vs epithelial Aldh1a1+ cell expansion, as only the stromal cells expanded during the estrous cycle. Also, RFP+ epithelial cells were not quantified during postpartum repair and visually appear to decrease (see comment below regarding Figure 5), so this statement is misleading.

The abstract was edited following the suggestions so that it depicts our results. Similarly, we have addressed the comment regarding Figure 5 and the interpretation of the post-partum regeneration experiments (see comment above for the full explanation and edits to the manuscript).

(3) Lines 65-69, 186-187: the authors should be clear when describing markers of putative epithelial vs mesenchymal stem/progenitor cells in the introduction (e.g., CDH2/SSEA1/SOX9 for epithelial and SUSD2 for mesenchymal).

Thank you for this suggestion. The Introduction section now states the following:

Lines 70-73: “These markers identify specific cell types in the endometrium, with *CDH2*^+^, *SSEA1*^+^, and *SOX9*^+^ cells corresponding to endometrial epithelial cells, while *SUSD2*^+^ cells corresponding to mesenchymal endometrial cells enriched in the perivascular regions (Cousins et al., 2021).”

(4) Lines 78-80: "Studies tracing the fate, ablation, and proliferative capacity of Lgr5+ cells in the uterus identified an Lgr5+ niche that is enriched in the crypts of the glandular epithelium and promotes endometrial regeneration (Seishima et al., 2019)." This statement is incorrect regarding regeneration, as Lgr5 marks stem/progenitor cells in the developing uterus but not the adult, during which endometrial regeneration occurs. Please revise.

Thank for you for this clarification. The statement has been revised and now reads as follows:

Lines 82-84: “Studies tracing the fate, ablation, and proliferative capacity of *Lgr5*^+^ cells in the uterus identified an *Lgr5*^+^ niche that marked stem/progenitor cells in the developing uterus (Seishima et al., 2019).”

(5) Recommend using free-form shapes to outline GE, LE, and EpSC in Fig 2C and stating in the text which clusters correspond to LE and GE (lines 167-169).

Thank you, this has been edited in Figure 2C and in the text, which now reads as follows:

Lines 176-177: “Clusters 5, 7, 21 and 16 were classified as glandular epithelial cells, and clusters 0, 2, 3, 10, 14, and 24 were classified as luminal epithelial cells (Figure 2C).”

(6) "Estrus" refers to the specific stage of the "estrous" cycle. Estrus cycle is incorrect and should be estrous cycle.

Thank you for pointing this out. It has been corrected throughout the manuscript.

**Reviewer #3 (Recommendations for the authors):**

(1) Suggest increasing the font size for some of the labels in the figures.

Thank you, we have increased font size in the figures to improve the quality.

(2) Need to include more details of the human endometrial tissue used in this study: pathology, age, and stage of menstrual cycle.

We thank the reviewer for the helpful suggestion. The details that are available to us have been included in Supplementary Table S5.

(3) Line 65-67 - Different endometrial stem cell subsets - SUSD2+ reside in perivascular regions, while other markers are located in glandular epithelium, need revision.

Thank you, this has been revised in the Introduction. The area now reads as follows:

Lines 70-73: These markers identify specific cell types in the endometrium, with *CDH2*^+^, *SSEA1*^+^, and *SOX9*^+^ cells corresponding to endometrial epithelial cells, while *SUSD2*^+^ cells corresponding to mesenchymal endometrial cells enriched in the perivascular regions (Cousins et al., 2021).

(4) Include a discussion about the interpretation of their current finding in relation to the dynamic regeneration observed in human endometrium due to menstrual bleeding/tissue breakdown, compared to the cycles of growth and regression that occur in mice.

This is a great suggestion. We have added the following statement to our Discussion section:

Lines 537-544: Additionally, our studies in human endometrium extend our characterization of ALDH1A1 as an adult endometrial stem cell marker and emphasize the importance of ALDH1A1+ in the regenerative potential of the endometrium. The conserved hormonal responses between human and mouse endometrium support the hypothesis that cycles of proliferation, differentiation, and regression, whether through resorption/autophagy in mice or menstrual breakdown in humans, are governed by concerted growth factor signaling and dedicated stem cell populations with the capacity to expand and differentiate across repeated cycles of repair. Our detailed studies in both mouse and human models indicate that ALDH1A1+ cells represent a dedicated cell type within the endometrium with the potential to drive repair during adulthood. Collectively, these findings advance our understanding of the mechanisms that control endometrial cycling and regeneration throughout the reproductive lifespan.

Reference

Ang, C.J., Skokan, T.D., and McKinley, K.L. (2023). Mechanisms of Regeneration and Fibrosis in the Endometrium. Annu Rev Cell Dev Biol 39, 197-221.

Bi, W.R., Jin, C.X., Xu, G.T., and Yang, C.Q. (2012). Bone morphogenetic protein-7 regulates Snail signaling in carbon tetrachloride-induced fibrosis in the rat liver. Exp Ther Med 4, 1022-1026.

Chen, M.Y., Zhao, F.L., Chu, W.L., Bai, M.R., and Zhang, D.M. (2023). A review of tamoxifen administration regimen optimization for Cre/loxp system in mouse bone study. Biomed Pharmacother 165, 115045.

Cousins, F.L., Murray, A., Esnal, A., Gibson, D.A., Critchley, H.O., and Saunders, P.T. (2014). Evidence from a mouse model that epithelial cell migration and mesenchymal-epithelial transition contribute to rapid restoration of uterine tissue integrity during menstruation. PLoS One 9, e86378.

Cousins, F.L., Pandoy, R., Jin, S., and Gargett, C.E. (2021). The Elusive Endometrial Epithelial Stem/Progenitor Cells. Front Cell Dev Biol 9, 640319.

Darzi, S., Werkmeister, J.A., Deane, J.A., and Gargett, C.E. (2016). Identification and Characterization of Human Endometrial Mesenchymal Stem/Stromal Cells and Their Potential for Cellular Therapy. Stem Cells Transl Med 5, 1127-1132.

Ghosh, A., Syed, S.M., Kumar, M., Carpenter, T.J., Teixeira, J.M., Houairia, N., Negi, S., and Tanwar, P.S. (2020). In Vivo Cell Fate Tracing Provides No Evidence for Mesenchymal to Epithelial Transition in Adult Fallopian Tube and Uterus. Cell Rep 31, 107631.

Huang, C.C., Orvis, G.D., Wang, Y., and Behringer, R.R. (2012). Stromal-to-epithelial transition during postpartum endometrial regeneration. PLoS One 7, e44285.

Khanmohammadi, M., Mukherjee, S., Darzi, S., Paul, K., Werkmeister, J.A., Cousins, F.L., and Gargett, C.E. (2021). Identification and characterisation of maternal perivascular SUSD2(+) placental mesenchymal stem/stromal cells. Cell Tissue Res 385, 803-815.

Kirkwood, P.M., Gibson, D.A., Shaw, I., Dobie, R., Kelepouri, O., Henderson, N.C., and Saunders, P.T.K. (2022). Single-cell RNA sequencing and lineage tracing confirm mesenchyme to epithelial transformation (MET) contributes to repair of the endometrium at menstruation. Elife 11.

Li, S.Y., Whiteside, S., Li, B., Sun, X., and DeFalco, T. (2025). Mesenchymal-to-epithelial transition of perivascular cells contributes to endometrial re-epithelialization. Nat Commun 16, 10174.

Niayesh-Mehr, R., Kalantar, M., Bontempi, G., Montaldo, C., Ebrahimi, S., Allameh, A., Babaei, G., Seif, F., and Strippoli, R. (2024). The role of epithelial-mesenchymal transition in pulmonary fibrosis: lessons from idiopathic pulmonary fibrosis and COVID-19. Cell Commun Signal 22, 542.

Patterson, A.L., Zhang, L., Arango, N.A., Teixeira, J., and Pru, J.K. (2013). Mesenchymal-to-epithelial transition contributes to endometrial regeneration following natural and artificial decidualization. Stem Cells Dev 22, 964-974.

Pimeisl, I.M., Tanriver, Y., Daza, R.A., Vauti, F., Hevner, R.F., Arnold, H.H., and Arnold, S.J. (2013). Generation and characterization of a tamoxifen-inducible Eomes(CreER) mouse line. Genesis 51, 725-733.

Rios, A.C., Fu, N.Y., Cursons, J., Lindeman, G.J., and Visvader, J.E. (2016). The complexities and caveats of lineage tracing in the mammary gland. Breast Cancer Res 18, 116.

Seishima, R., Leung, C., Yada, S., Murad, K.B.A., Tan, L.T., Hajamohideen, A., Tan, S.H., Itoh, H., Murakami, K., Ishida, Y., et al. (2019). Neonatal Wnt-dependent Lgr5 positive stem cells are essential for uterine gland development. Nat Commun 10, 5378.

Zeisberg, M., Shah, A.A., and Kalluri, R. (2005). Bone morphogenic protein-7 induces mesenchymal to epithelial transition in adult renal fibroblasts and facilitates regeneration of injured kidney. J Biol Chem 280, 8094-8100.
